# Functional Brain Network Mechanism of Hypersensitivity in Chronic Pain

**DOI:** 10.1038/s41598-017-18657-4

**Published:** 2018-01-10

**Authors:** UnCheol Lee, Minkyung Kim, KyoungEun Lee, Chelsea M. Kaplan, Daniel J. Clauw, Seunghwan Kim, George A. Mashour, Richard E. Harris

**Affiliations:** 1 Department of Anesthesiology, University of Michigan Medical School, Ann Arbor, MI 48109 USA; 2 Center for Consciousness Science, University of Michigan Medical School, Domino’s Farms, P.O. Box 385, Ann Arbor, MI 48105 USA; 3 Neuroscience Graduate Program, University of Michigan, Ann Arbor, MI USA; 4 Chronic Pain and Fatigue Research Center, University of Michigan, Ann Arbor, MI 48105 USA; 5 Department of Physics, Pohang University of Science and Technology (POSTECH), Pohang, South Korea

## Abstract

Fibromyalgia (FM) is a chronic widespread pain condition characterized by augmented multi-modal sensory sensitivity. Although the mechanisms underlying this sensitivity are thought to involve an imbalance in excitatory and inhibitory activity throughout the brain, the underlying neural network properties associated with hypersensitivity to pain stimuli are largely unknown. In network science, explosive synchronization (ES) was introduced as a mechanism of hypersensitivity in diverse biological and physical systems that display explosive and global propagations with small perturbations. We hypothesized that ES may also be a mechanism of the hypersensitivity in FM brains. To test this hypothesis, we analyzed resting state electroencephalogram (EEG) of 10 FM patients. First, we examined theoretically well-known ES conditions within functional brain networks reconstructed from EEG, then tested whether a brain network model with ES conditions identified in the EEG data is sensitive to an external perturbation. We demonstrate for the first time that the FM brain displays characteristics of ES conditions, and that these factors significantly correlate with chronic pain intensity. The simulation data support the conclusion that networks with ES conditions are more sensitive to perturbation compared to non-ES network. The model and empirical data analysis provide convergent evidence that ES may be a network mechanism of FM hypersensitivity.

## Introduction

Fibromyalgia (FM) is a disorder characterized by widespread musculoskeletal pain accompanied by fatigue, sleep, memory and psychological disturbance^1,2^. Individuals with FM have alterations in brain structure, function, and neurochemistry that are thought to lead to the hyperalgesia and chronic pain commonly reported in this condition^3–6^. Previous electroencephalogram (EEG) studies have also shown differences in power spectra obtained from FM patients and pain-free controls, suggesting alterations in brain network function such as thalamocortical dysrhythmia^7–9^. Along with this theory, increased hyper-excitability or hyperactivity of the nociceptive system has been proposed as a potential mechanism of clinical pain in FM^4,10–13^. There have been no explicit studies that explain, from a large-scale brain network perspective, how these objective signals interact to engender the subjective sensation of chronic pain.

Hypersensitive responses to external stimuli, as displayed in FM patients, have been widely observed in various physical and biological systems such as cascade failures in a power-grid, abrupt state transitions in an electronic circuit and chemo-mechanical systems, epileptic seizures in the brain, and the sensitive frequency detection of the cochlea^14–17^. These systems all have a common characteristic whereby a small perturbation gives rise to explosive and global propagation in the system. A network property known as “explosive synchronization” (ES) has been studied as the underlying mechanism of abrupt state transitions within these types of systems and described as a discontinuous transition from an incoherent state to a synchronized state^18–24^. In a network that displays ES condition(s), a perturbation rapidly propagates across the whole network through synchronization. Our past work suggests that ES conditions are not present in the resting state of normal human brains^25^. We hypothesized that ES may be a mechanism of FM hypersensitivity, with the expected result that FM patients with an enhanced ES condition would have greater network sensitivity and increased chronic pain.

**Figure 1:**
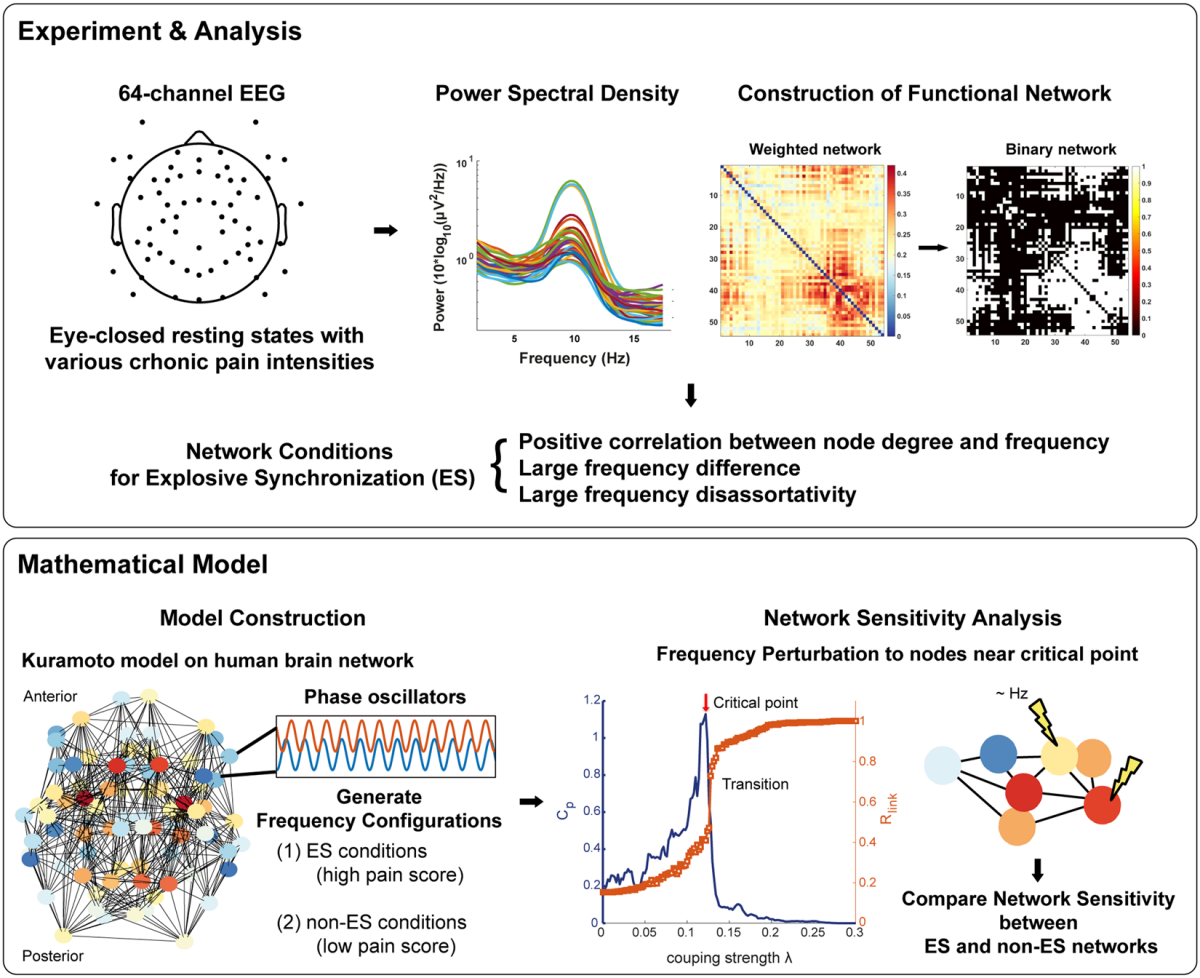
Schematic diagram of study design. The study was composed of two parts, an experimental analysis section and a mathematical modeling section. The experimental phase was carried out by recording 64-channel electroencephalogram (EEG) of fibromyalgia (FM) patients. A functional network was constructed with the weighted phase lag index (WPLI) of the EEG signal, and power spectral density analysis was performed to obtain a node degree and frequency for each EEG channel. We then confirmed the relationship between the three ES conditions and the intensity of chronic pain. For the mathematical phase of the study, we generated a human brain network model based on the Kuramoto model and diffusion tensor imaging (DTI). The frequency configurations with ES and non-ES conditions were considered as the brain network states of high and low pain scores, respectively. These were used to explore the network sensitivity of ES and non-ES networks as tested with a frequency perturbation near the critical point.

To test this hypothesis, we first analyzed the resting-state EEG network configurations of 10 FM patients and assessed (1) whether the FM brain network displayed characteristics of ES conditions (not found in normal humans), and (2) the correlation between the strength of ES conditions and chronic pain intensity (Fig. 1). For the ES conditions, we examined positive degree frequency correlation, large frequency difference, and large frequency disassortativity (a tendency of higher frequency nodes to link with lower frequency nodes or vice versa) between linked nodes in the networks^18–24^. These are typical network conditions shown to suppress gradual synchronization, suddenly triggering global synchronization around a critical point. Second, we tested whether these ES conditions produce hypersensitive network characteristics in response to stimulation. This network sensitivity was quantitatively compared between the human brain networks with ES and non-ES conditions using a frequency perturbation.

We demonstrate for the first time that the FM brain displays ES conditions. Furthermore, the degree of the ES conditions significantly correlated with the intensity of chronic pain. With computational models, we also demonstrate that brain networks with ES conditions are hypersensitive to a frequency perturbation. We conclude that ES may be a network mechanism underlying the hypersensitivity of the FM brain.

## Materials and Methods

### Participants and experiment protocol

This study was performed at the University of Michigan. All study participants gave written informed consent, and the study protocol and informed consent documents were approved by the University of Michigan Institutional Review Board (Ann Arbor, Michigan). We confirmed that all methods were performed in accordance with the relevant guidelines and regulations. 17 FM patients (all females, age mean ±SD: 45.7 ± 11.4) were recruited as part of a phase 2 clinical trial (NCT01914679) evaluating a novel noninvasive brain stimulation device (Reduced Impedence Noninvasive Cortical Electrostimulation, RINCE) as a treatment for chronic pain. For this study, we analyzed only baseline EEG data, before any treatments occurred. Ten out of seventeen patients’ data were used for the analysis. Seven subjects were excluded due to missing EEG data or high electromyogram or ocular movement artifact. Inclusion criteria were: FM patients who met the 1990 ACR diagnostic criteria for FM, female, and between the age of 18–65. Exclusion criteria were: current psychiatric disorder, history of suicide attempt in preceding 5 years, any history of bipolar disorder, schizophrenia or other psychotic disorder, a Hospital and Anxiety Depression score greater than 11 for either anxiety or depression, other chronic infection or condition that may cause pain, history of seizure disorder, pregnant or breastfeeding, history of alcohol or drug abuse, BMI greater than 40 kg/m^2^, or the use of stimulant medication, centrally active analgesics, or anesthetic or narcotic patches.

### EEG recording

64-channel sensor net from Electrical Geodesics, Inc. (Eugene, OR) was used to acquire the EEG data with a sampling frequency of 500 Hz and the electrode impedance was kept below 50 KΩ.

The EEG protocol consisted of 10 minutes of resting state (5 minutes eyes open, 5 minutes eyes closed). Clinical pain was assessed immediately before the resting state period with a Visual Analog Scale (VAS) where “0” represented no pain and “100” represented the worst pain imaginable.

Noise and artifacts of the signals were automatically removed by the EEGLAB toolbox based on the power spectrum with a 10 dB threshold. In addition to the automatic rejection, we visually inspected the data and excluded artifacts. Independent component analysis (ICA) was also applied to the signals (function runica.m, EEGLAB, MATLAB toolbox, https://sccn.ucsd.edu/eeglab/index.php) and removed components of eye movements, cardiac signals, and focal noise. Noisy channels were also removed with a high-pass filter from 0.5 Hz, and 4-minute eyes closed artifact-free resting-state EEG signals were re-referenced to an average reference. We focused our analysis on the alpha frequency range (8–13 Hz) because it is the dominant peak frequency in the eyes-closed resting state.

### EEG network analysis

To reconstruct the functional brain network, we used the weighted phase lag index (WPLI)^26^, which is a measure that captures phase locking between two signals with high robustness to volume conduction.

1 $$WPL{I}_{ij}=\,\frac{|E\{\Im ({C}_{ij})\}|}{E\{|\Im ({C}_{ij})|\}}=\,\frac{|E\{|\Im ({C}_{ij})|sgn(\Im ({C}_{ij}))\}|}{E\{|\Im ({C}_{ij})|\}}$$

where ℑ(*C*_*ij*_) is an imaginary part of cross-spectrum *C*_*ij*_ between two signals *i* and *j*. The cross-spectrum *C*_*ij*_ is defined as ${Z}_{i}{Z}_{j}^{\ast }$, where a complex value *Z*_*i*_ denotes the Fourier spectra of the signal *i* for a particular frequency and ${Z}_{j}^{\ast }$ is the complex conjugate of *Z*_*j*_. *C*_*ij*_ can be represented as *Re* ^*iθ*^, where *R* is magnitude and *θ* is the relative phase between signal *i* and *j*. If the phases of one signal *i* always lead or lag those of the other signal *j*, then *WPLI*_*ij*_ equals 1. On the other hand, if the phase lead/lag relationship of two signals is perfectly random, the *WPLI*_*ij*_ value is 0.

We segmented the EEG signals into 10-s epochs with 2-s overlap. WPLI matrix was obtained for each 10-s window. We then took the average of WPLI matrices over all windows. A binary adjacency matrix A_ij_ was constructed using the top 40% of averaged WPLI values among all channel pairs. If the *WPLI*_*ij*_ was included in the top 40% of WPLI values, then A_ij_ = 1, otherwise, A_ij_ = 0. We then calculated the node degree, which is the number of connections of each node in the network. We tested several thresholds: 30, 40, 50, 60, and 70%; the analysis results are robust below 50% (See Supplementary Fig. S1).

### EEG network configuration for ES conditions

First, we calculated the Spearman correlation between node degree and frequency, which has been identified as one of the network conditions for ES^18^. For each 10-s window, we calculated the power spectral density (PSD) for all channels (“pwelch.m” in MATLAB, with 2-s Hamming windows, 1-s overlaps, and frequency resolution = 0.2 Hz). At each window, we obtained the frequency that has median power within a frequency band. We took the average of the median frequency over all windows to calculate the correlation between node degree and frequency.

Next, the frequency difference Y_ij_ and the frequency assortativity A_f_ between connected nodes were calculated to investigate the functional network conditions for ES^19,21^. The frequency difference Y_ij_ is defined as

2 $${Y}_{ij}=\frac{|{Y}_{i}-{Y}_{j}|}{{Y}_{i}+{Y}_{j}}$$

where Y_i_ and Y_j_ are the median frequencies of the signal *i* and *j*, respectively. We used the average of frequency difference among connected nodes, <Y_ij_> to evaluate the frequency difference of one individual. The frequency assortativity A_f_ is a Spearman correlation between the node frequencies and the average frequencies of connected neighbors in a network. If A_f_ < 0, the higher frequency nodes tend to link with lower frequency nodes, or vice versa, which indicates that the frequency relationship between nodes in the network is diassortative. Furthermore, Spearman correlations were calculated to investigate the relationship between clinical pain scores and network sensitivities represented by the degree-frequency correlation, frequency difference and frequency assortativity for all subjects.

Lastly, we calculated the Spearman correlation between the pain score and the degree/median frequency of each channel for all patients. This was performed to determine which brain areas were associated with the pain score. We calculated z-scores of degree/median frequency over all channels for each subject. With the z-scores of each channel for all patients, we calculated the correlation between the pain scores and those z-scores. We considered a p-value less than 0.05 as statistically significant.

### Human brain network models

A simple coupled oscillator, the Kuramoto model, was used to simulate the interaction between brain areas in the human brain network constructed with DTI of 82 nodes including cortical and subcortical areas^27^.

3 $$\frac{{\rm{d}}{\theta }_{i}}{{\rm{d}}t\,}={\omega }_{i}+{\sum }_{j=1}^{N}{\lambda }_{ij}{A}_{ij}\,\sin ({\theta }_{j}-{\theta }_{i})\,$$

where *N* is the total number of oscillators, *θ*_*i*_ and *ω*_*i*_ are the phase and natural frequency of the oscillator *i*, respectively, *i* = 1, 2, …, *N* at time *t*. For simplicity, we assumed that the coupling strength between oscillators *i* and *j*, *λ*_*ij*_ is constant and uniform, i.e., λ_ij_ = *λ*. A_ij_ is the connection matrix of the human DTI anatomical network. A_ij_ = 1 if oscillators *i* and *j* are connected, and A_ij_ = 0 if they are not. Each oscillator *i* is assigned a random initial phase *θ*_*i*_, uniformly distributed between [−π, π] and a random initial frequency *ω*_*i*_, drawn from some arbitrary distribution function g(ω). The oscillators become spontaneously synchronized if *λ* is larger than a certain critical value.

We designed the natural frequency configurations *g*(*ω*) to embody the network conditions that were consistent with the empirical results of patients with varying pain scores. Specifically, empirical data were used to generate ES and non-ES networks as follows:

i) We applied the perturbations to the highest degree nodes (1, 5, 10, 15, and 20). Here we only present the significant perturbation results of the 15 highest degree nodes (~18% of all nodes), which includes brain regions such as the putamen, insula, precuneus, thalamus, superior frontal and superior parietal regions^27^. The perturbation results and the names of the brain regions for the other highest degree nodes are presented in Supplementary Fig. S2.
ii) The values of *ω*_*i*_ that were not involved in the 15 highest degree nodes were obtained from the Gaussian distribution function $g(\omega )={G}_{\bar{\omega },{\sigma }_{\omega }}(\omega )$ with the mean $\bar{\omega }=10$ Hz, deviation σ_*ω*_ = 0.2 Hz, mimicking the alpha rhythms of human EEG activity^28^.
iii) The 15 highest degree nodes were divided by two subgroups α and β in which oscillators have higher and lower frequency ranges than average – i.e., ${G}_{\overline{{\omega }_{\alpha }},{\sigma }_{{\omega }_{\alpha }}}({\omega }_{\alpha })$ with $\overline{{\omega }_{\alpha }}=10.2$ Hz, ${{\rm{\sigma }}}_{{\omega }_{\alpha }}=0.02$ Hz and ${G}_{\overline{{\omega }_{\beta }},{\sigma }_{{\omega }_{\beta }}}({\omega }_{\beta })$ with $\overline{{\omega }_{\beta }}=9.8$ Hz, ${{\rm{\sigma }}}_{{\omega }_{\beta }}=0.02$ Hz for each subgroup, respectively − causing large frequency differences as well as forming a V-shape relationship between the node degree and frequency in the network, which facilitates ES^19^.
iv) With the above conditions, we carried out the simulation of 100 configurations with positive degree-frequency correlation, frequency difference >0.01, and frequency assortativity <−0.25 as the high pain score condition, determined by empirical observation of the participants who had a pain score >42. We refer to those network conditions as the ES conditions.
v) We also generated the frequency configurations that have Gaussian distribution function $g(\omega )={G}_{\bar{\omega },{\sigma }_{\omega }}(\omega )$ with the mean $\bar{\omega }=10$ Hz, deviation σ_*ω*_ = 0.2 Hz. The 100 configurations with the frequency assortativity >−0.25 were considered to be the low pain score conditions. We deem these as non-ES conditions.

The collective dynamics of the ensemble of the oscillators were measured by the order parameter,

4 $$z(t)=r(t){e}^{-i{\psi }(t)}\equiv \frac{1}{N}{\sum }_{j=1}^{N}{e}^{-i{\theta }_{j}(t)}$$

where *ψ*(*t*) is the average global phase. The modulus *r*(*t*) = |*z*(*t*)| so-called order parameter represents the degree of synchrony, being equal to 0 when the oscillators’ phases are uniformly distributed in [0, 2*π*) and 1 when they all have the same phase. The level of phase synchronization is determined by a time average of the order parameter after a transient period *T*_Δ_ = 5000, R_linked_ = 〈*r*(*t*)〉_*T*_, with the whole time period *T* = 10000. We observe the state of the network by increasing the coupling strength λ by δλ = 0.002, from λ = 0.

### Network sensitivity analysis

We introduced a frequency perturbation into the Kuramoto model slightly below the critical point to simulate external stimuli. We used the pair correlation function *C*_*p*_, which has been used to quantify the susceptibility of statistical physics models^29–31^ to measure network sensitivity at a global level as well as to find the critical point of the network. The pair correlation function in the Kuramoto model is defined as,

5 $${C}_{p}=N\{{\langle R{e}^{2}[z(t)]\rangle }_{t}-{\langle Re[z(t)]\rangle }_{t}^{2}\},$$

where *N* and *z*(*t*) are the total number of oscillators and complex order parameter, respectively. *Re*[*z*(*t*)] is the real part of *z*(*t*) in Eq. (4).

The pain-related brain regions such as insula, precuneus, superior frontal cortices, parietal cortices and the thalamus^4,5,32,33^ were perturbed as the target sites of the human brain network. The median frequency of the network frequency configuration was given to the target sites to facilitate ES.

The network sensitivity Δ(*C*_*p*_) was defined as the absolute difference between the pair correlation functions at the critical coupling strength *λ*_*c*_, before and after the frequency perturbation as follows,

6 $${\rm{\Delta }}({C}_{p})\equiv {|{C}_{p}({s}_{p})-{C}_{p}({s}_{0})|}_{\lambda ={\lambda }_{c}}$$

where *C*_*p*_(*s*_0_) and *C*_*p*_(*s*_*p*_) are the pair correlation functions before and after the frequency perturbation, respectively. The statistical difference of the network sensitivities between ES and non-ES networks was obtained by the sign test (p-value < 0.001 was considered as a significantly higher sensitivity in ES network).

## Results

### EEG network properties in FM patients

**Figure 2:**
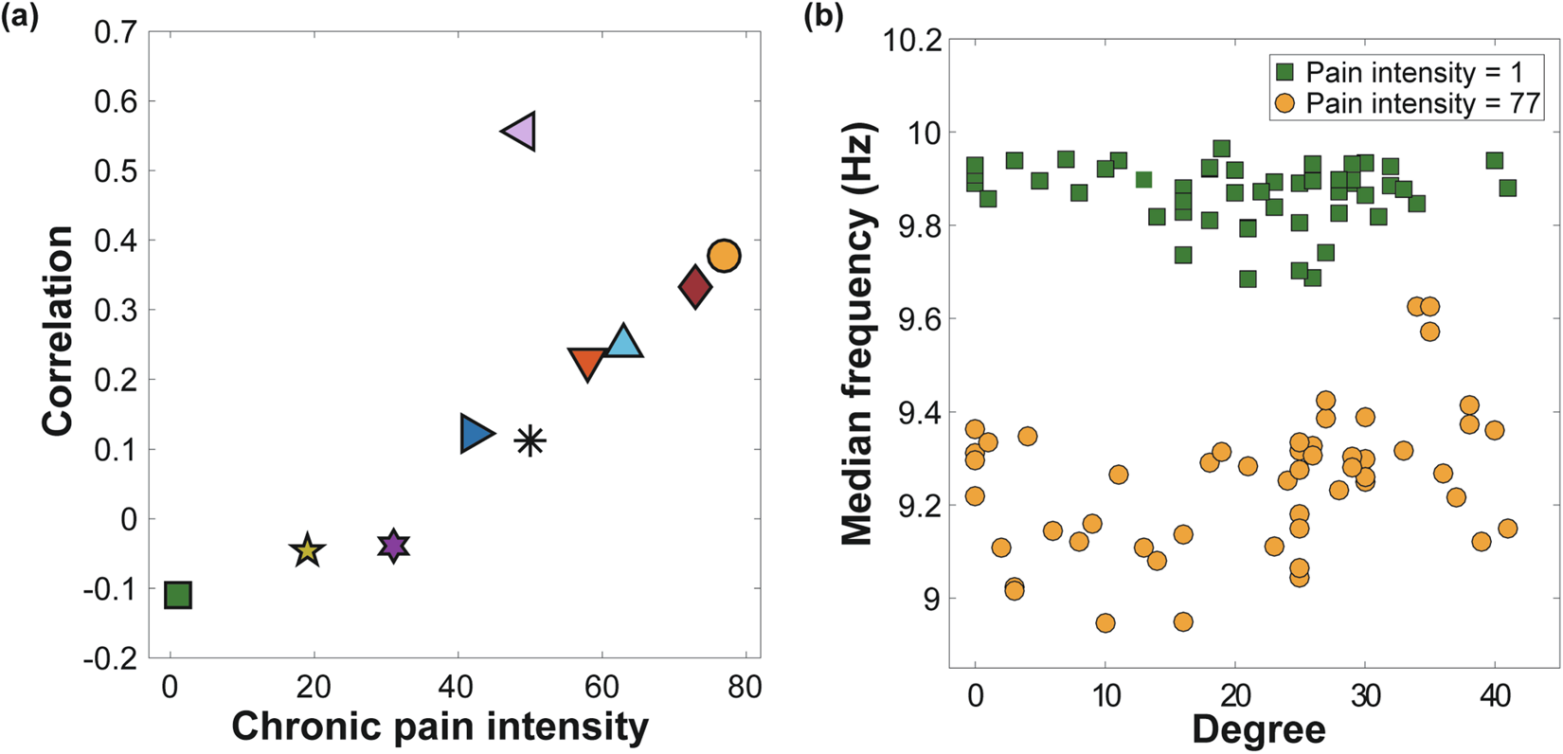
Node degree and frequency are positively correlated with chronic pain intensity. (**a**) The degree-frequency correlation coefficient positively correlates with the pain intensity. Each marker represents an individual. (**b**) The relationship between node degrees and median frequencies for all EEG channels from two exemplary subjects with low (1) and high (77) pain intensities. The individual with a high pain score displayed a positive relationship between node degree and median frequency of EEG channels, whereas no correlation is observed for the individual with low pain. Each circle and square represents an EEG channel.

Figure 2(a) illustrates the relationship between the clinical pain intensities and the correlation coefficients between node degree and frequency for all subjects. A positive correlation between node degree and frequency is known to be one of the network conditions for ES in a scale-free network^18^. In the EEG data shown in Fig. 2(a), participants with pain scores higher than 42 had a tendency to have a positive correlation between node degree and frequency, suggesting conditions for ES. By contrast, the other subjects had negative correlations. Pain intensities of FM patients were positively correlated with the correlation coefficient between node degrees and median frequencies (Spearman correlation = 0.79, p < 0.01).

Figure 2(b) presents the exemplary cases of two individuals, one with the lowest and the other with the highest chronic pain intensity. For the FM patient with the highest pain rating (77 out of 100), nodes with larger degree also had higher frequencies (Spearman correlation = 0.38, p < 0.01). By contrast, the FM patient with the lowest paint intensity (=1) shows low correlation between node degrees and frequencies (Spearman correlation = −0.11, p > 0.05).

**Figure 3:**
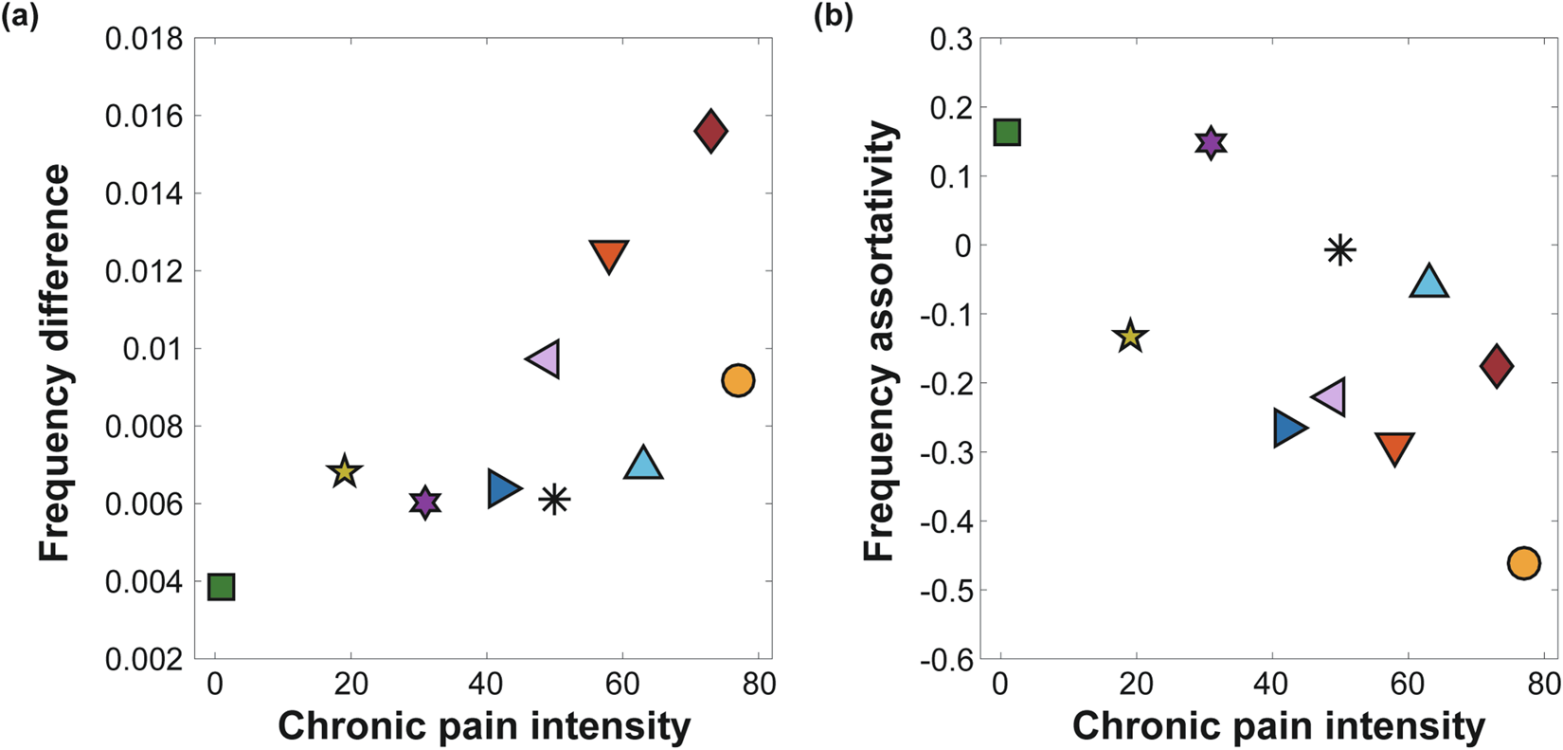
Frequency difference and frequency assortativity are correlated with chronic pain intensity. (**a**) Frequency difference and (**b**) frequency disassortativity (negative assortativity) among EEG channels correlate with the pain scores. Each marker represents an FM patient.

The frequency difference and the frequency assortativity of ten FM patients are presented with their associated chronic pain intensity rating in Fig. 3(a) and (b). A large frequency difference is another ES condition^19,21^ along with the positive degree-frequency correlation. Chronic pain intensity was significantly correlated with frequency difference between linked nodes in the network (Spearman correlation = 0.72, p < 0.05).

Large frequency disassortativity is another condition for ES identified in model networks^23^. Frequency disassortativity is associated with frequency difference, but it accounts for the anti-correlation between the frequencies of linked nodes rather than just the absolute frequency difference. FM patients with greater chronic pain (>42) had frequency disassortativity wherein higher frequency nodes tended to link with lower frequency nodes. The opposite was observed for FM patients with lower chronic pain intensities (<42), where higher frequency nodes tended to link with higher frequency nodes (assortativity). The correlation between chronic pain intensity and frequency assortativities for the ten FM patients was −0.59 (p = 0.08).

We observed large correlations of the degree-frequency correlation coefficient, frequency difference, and frequency assortativity with chronic pain intensity in the ten FM patients. However, the ES conditions were defined by the global relationship between structure (node degrees) and function (frequencies) of the EEG network, rather than the node degree and frequency of each EEG channel. It is notable that the ES conditions were observed only in alpha band (8–13 Hz), whereas the other frequency bands: delta (0.1–4 Hz), theta (4–8 Hz), beta (13–25 Hz), and gamma (25–45 Hz), did not show any significant ES conditions (see Supplementary Fig. S3 for the other frequency bands). Here, we measured which channel’s node degree or frequency is correlated with chronic pain, to identify the regional node degree and frequency attributes of the FM patients with high and low pain intensities.

### Regional network properties with pain intensity

**Figure 4:**
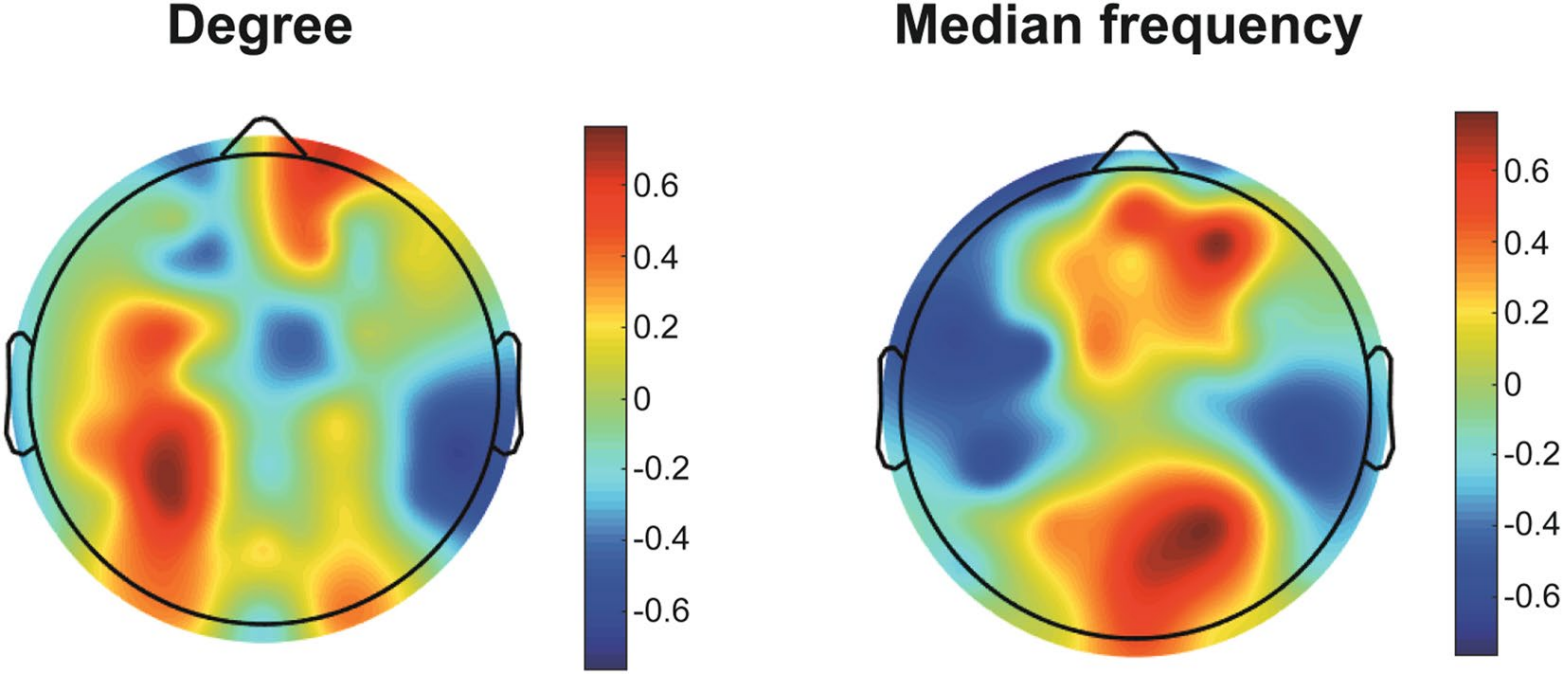
The relationships of regional node degree (Left) and median frequency (Right) with pain intensity. The correlation coefficients between z-values of node degrees (median frequencies) and pain scores for the ten FM patients and each EEG channel. The colour bar represents Spearman correlation coefficient from −1 to 1.

Figure 4 shows the Spearman correlation coefficients between the pain intensity and the z-values of node degree and median frequency. For the node degree, the left posterior area shows a correlation with the pain intensity (Spearman correlation = 0.66 with p < 0.05). For the median frequency, the anterior and posterior regions demonstrate consistent large correlations with chronic pain intensity (Spearman correlation = 0.74 with p < 0.05 and 0.72 with p < 0.05, respectively). These data indicate that higher pain scores have higher median frequencies in anterior and posterior regions. This regional frequency attribute of the FM patients may contribute to ES network configurations such as positive degree-frequency correlation, negative frequency-frequency correlation, and large frequency difference.

### ES and non-ES network conditions

**Figure 5:**
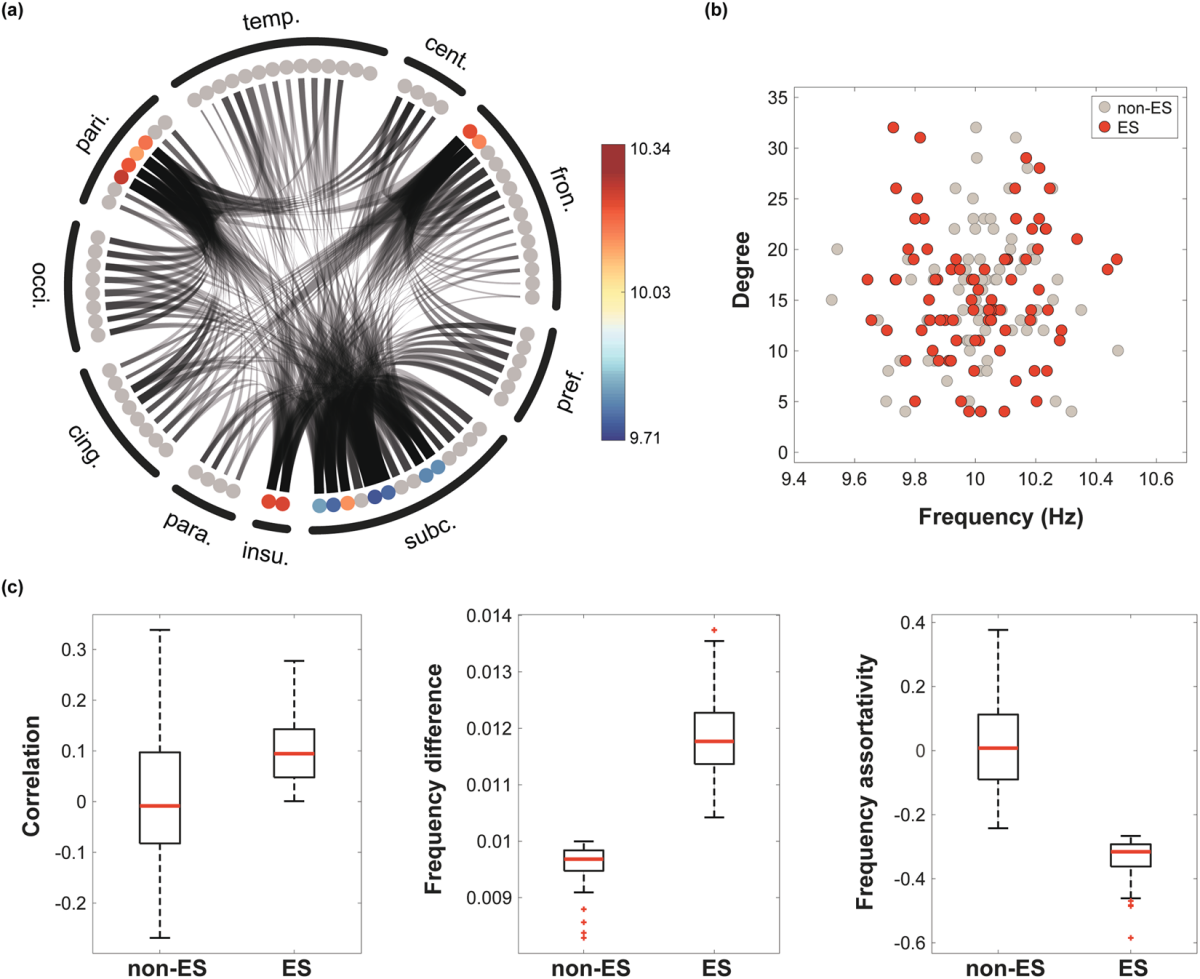
Network configurations of ES and non-ES conditions. (**a**) A frequency configuration in the human brain network consisting of 82 brain regions. The asymmetric frequency distributions of the top 15 hub nodes are denoted with different colors. Each circle corresponds to a brain region, and the dark/gray lines are the connections among the regions. 82 regions are clustered into 10 larger brain regions, denoted in Supplementary Table S1. (**b**) The relationships between node degrees and frequencies for the ES and non-ES conditions. The ES condition has a V-shape relationship, in which the hub nodes have large and small frequencies suppressing synchronization until a critical point (the key mechanism of ES), whereas the non-ES condition has a random relationship. (**c**) The brain network model with ES conditions has positive degree-frequency correlation coefficient, larger frequency difference, and negative frequency assortativity compared to non-ES condition. Red solid lines throughout the panels are medians of 100 frequency configurations.

We established two anatomically informed human brain network models, one with ES and another with non-ES conditions, to test whether the human brain with ES conditions is associated with hypersensitivity to stimuli. Because it is known that the network topology significantly influences the network susceptibility^18^, we needed to quantitatively test the network sensitivity with an external perturbation to human brain networks with states near critical points for both ES and non-ES conditions. Considering the network structure influence, Fig. 5(a) presents an example of frequency configuration used in an ES network model to simulate the EEG networks associated with higher pain intensity. Frequency mismatches between nodes were accomplished by having one subgroup of high degree nodes (L. caudate, L. and R. precuneus, superior frontal, superior parietal and insula) with higher frequencies and another subgroup of high degree nodes (L. and R. thalamus, putamen and hippocampus) with lower frequencies (see Materials and Methods); this mismatch created network conditions favoring ES. The relationship between the frequency and degree thereby forms a V-shape causing large frequency differences among the hub nodes as shown in Fig. 5(b) for the ES configuration (red circles). The property of frequency disassortativity for ES networks can be also naturally attributed by the V-shape relationship between frequency and node degree. Figure 5(c) demonstrates the network configurations we used for both ES and non-ES network models, including degree-frequency correlation, frequency difference, and frequency assortativity. The ES network has positive degree-frequency correlation, large frequency difference (>0.01), and large frequency disassortativity (<−0.25). These conditions do not exactly match with the EEG data, but we simulated the essential factors to identify the influence on network sensitivity in the complex and hierarchical human brain network.

### Network sensitivity

**Figure 6:**
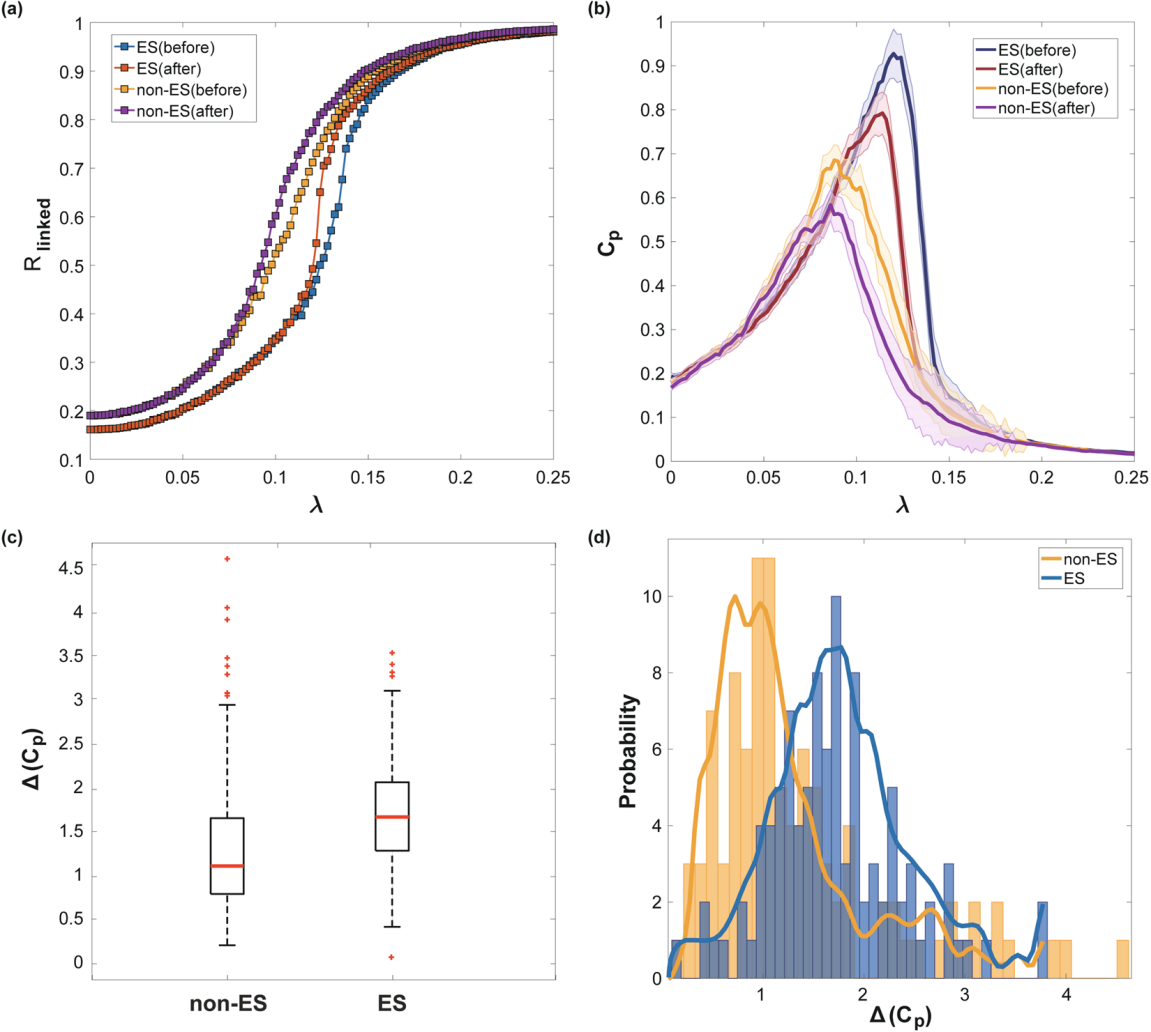
The comparison of the human brain networks of ES and non-ES conditions after frequency perturbations in hubs nodes (insula, precuneus, superior frontal cortices, parietal cortices with thalamus). (**a**) Median synchronization level ***R***_***linked***_ vs. coupling strength ***λ*** of networks for ES and non-ES conditions before and after the perturbation. 100 different frequency configurations were implemented for the median ***R***_***linked***_. The ES condition shows a steeper synchronization, and the perturbation induces larger synchronization. (**b**) Brain network susceptibility ***C***_***p***_ vs. coupling strength ***λ*** for ES and non-ES conditions before and after the perturbation. ES conditions have larger ***C***_***p***_ than non-ES conditions. The perturbation induces larger synchronization, which is measured as reduced ***C***_***p***_ and with larger alternation of ***C***_***p***_ in ES. The thick lines and shaded region indicate median **C**_**p**_ and standard error over 100 configurations. (**c**) The brain network containing the ES conditions shows significantly larger network sensitivity **Δ**(***C***_***p***_) (p < 0.05), despite the large variances. The red line denotes the median **Δ**(***C***_***p***_) over 100 configurations. (**d**) The variance of **Δ**(***C***_***p***_) indicates the dependency of **Δ**(***C***_***p***_) on the network configurations within each ES and non-ES condition. The blue (yellow) bar illustrates the number of network configurations with the same **Δ**(***C***_***p***_). The blue (yellow) thick line indicates a fitted line for the distributions of **Δ**(***C***_***p***_).

We tested the sensitivity of human brain network models with ES and non-ES conditions. First, we assumed that the human brain in the resting state resides near the critical point of state transition, in which functional brain networks have the most complex interaction pattern, as well as the largest *C*_*p*_^34^. The state of the network was defined by the synchronization level across brain regions. A frequency perturbation was introduced at the critical point found for each network configuration, and the sensitivity of ES and non-ES brain network configurations was quantified. Figure 6(a) demonstrates the change of network synchronization R_linked_ with increasing coupling strength (the only control parameter of the model). The ES and non-ES conditions produce distinctive synchronization patterns (Fig. 6a). The non-ES networks synchronize gradually, whereas the ES networks demonstrate delayed but steep synchronization. With the frequency perturbation, the ES network was more abruptly synchronized with a larger change of R_linked_ than the non-ES network.

Figure 6(b) demonstrates the pair correlation function value *C*_*p*_ of ES and non-ES networks before and after the frequency perturbation. The critical coupling strength λ_*c*_ for each network configuration was determined by the largest *C*_*p*_, which mirrors the most variable correlation pattern for a network. In comparison, the ES networks induce larger decreases of *C*_*p*_ than non-ES networks. This decreasing correlation function implies that the ES network gives rise to a more global propagation of synchronization from the perturbed node, strongly entraining different frequencies regionally distributed in the network.

Figure 6(c) shows the network sensitivity defined as the change of *C*_*p*_ with the frequency perturbation. The ES network has a significantly larger network sensitivity Δ(C_p_) than the non-ES network (p < 0.001, sign test). The median sensitivity values of ES and non-ES networks were 1.08 and 1.64, respectively. The distributions of network sensitivity over 100 configurations for each network are shown in Fig. 6(d). The results suggest that if the brain network has an ES condition, the brain may be highly sensitive to stimuli.

## Discussion

In this study, we hypothesized that chronic pain is associated with a highly sensitive functional brain network that results from conditions favoring rapid synchronization. From the empirical data, we found evidence that FM patients have brain network configurations with conditions for ES. Furthermore, there is a positive correlation between conditions for ES and the degree of clinical pain. These empirical findings support our hypothesis that ES can be a potential mechanism of hypersensitivity in the FM brain network. We also examined the specific brain regions that may subserve chronic pain by observing higher correlations between pain intensity and node degrees within posterior nodes and median frequencies in anterior and posterior regions. Although EEG lacks spatial resolution compared with other neuroimaging modalities, higher frequencies of anterior and posterior regions seemed to be related to chronic pain intensity^4^.

Theoretically, the ES network could exhibit increased sensitivity to a specific external perturbation, but this has yet to be directly assessed in a network. Therefore, we tested whether an anatomically informed human brain network with ES conditions would be differentially sensitive to external stimuli. The brain networks generated had a complex modular structure, which can inhibit the occurrence of ES^17^. The comparison between ES and non-ES conditions demonstrated that the ES conditions can give rise to higher sensitivity to the stimulation even in a complex human brain network. These results suggest that ES may be one possible network mechanism involved in the hypersensitivity to external stimuli in FM brain networks.

Our findings of enhanced ES in FM may have relevance to previous EEG studies. Vanneste *et al*. found altered alpha power in FM patients as compared to pain free controls^9^. We also observed ES conditions specifically within the alpha frequency band in FM. Frequency-degree relationships within the alpha frequency band that are indicative of ES may play a role in the varying degrees of pain across individuals. Although not specifically tested here, the previously proposed thalamocortical dysrhythmia^7^ may also play a role in ES, as the thalamus was a sensitive node to stimulation in our brain network models. We also note that our findings are specific to the alpha frequency band as other bands did not show degree-frequency relationships with pain. This is in agreement with a lack of consistent EEG findings in other (non-alpha) frequency bands within FM^7–9^.

ES is a general phenomenon in nature that has been studied in physics since 2011 and that explains various physical and biological state transitions^18–24^. Recently, we identified ES conditions and abrupt synchronization transitions in human brain networks during the lightly anesthetized state^25^. As the anesthetic concentration crossed the threshold of unconsciousness, ES conditions developed in human brain networks, facilitating frequent and sudden state transitions between conscious and unconscious states. That study demonstrated for the first time that the network conditions for ES are present in empirically derived functional brain networks. Another computational model study demonstrated that, even though highly clustered brain networks inhibit ES, slight changes of network cluster structures facilitated the transition to a seizure state in the brain^17^. In addition, a recent model study showed that ES enhances frequency selectivity and signal-to-noise ratio of the cochlea^16^. These recent empirical and computational model studies indicate that ES can, indeed, take place during diverse state transitions in the brain.

In this study, we hypothesized that the hypersensitivity of the central nervous system in FM patients is associated with an altered brain network configuration that displayed greater ES conditions. Our computational simulation generated both normal (non-ES) and abnormal (ES) brain networks. Networks with ES conditions were susceptible to perturbation at a critical point as expected and we assumed that both brain networks in the resting state may be residing near the critical points determined by the given network configurations. However, the brain network under ES conditions showed relatively abrupt state transitions accompanied by a large change of synchronization level. In addition, a perturbation immediately influenced global brain regions through synchronization in the ES network. The ES was manifested as a discontinuous transition from an incoherent state to synchronized state when a small perturbation was introduced into to the system around the point of criticality.

With respect to treatment of FM, we expect that our study may ultimately suggest new approaches for analgesic treatments. ES provides a theoretical framework and quantitative approach to test interventions that shift a hypersensitive brain network to a more normal brain network. This approach is conceivable, as Zhang *et al*. reported that an ES network can be modified into a non-ES network by inhibiting a small fraction of network activities^22^. Considering the characteristic hierarchical hub structure of the human brain network, it may be possible to convert an ES network to a non-ES network just by modulating one or two hub nodes. Indeed, transcranial magnetic stimulation and/or transcranial direct current stimulation (both interventions shown to modulate chronic pain in FM) may be improved by “targeting” these sensitive hub nodes^35,36^; the application of deep brain stimulation to critical nodes that could modify ES conditions is another therapeutic possibility that could be explored.

### Limitations

Our empirical data analysis and model study have several limitations. First, we did not use normal subjects as controls. Instead, we recruited the FM patients with various pain intensities ranging from 1 to 77. The FM patients with less pain (<42) were similar to our past study of healthy subjects with respect to their resting state data. In other words, we studied a subpopulation that did not have conditions for ES but future study with comparison to healthy controls is warranted. Second, scalp EEG assesses superficial brain activity and thus the empirical EEG analysis results cannot be directly compared with the diffusion tensor imaging (DTI) based network model that included cortical and subcortical areas. Magnetoencephalography or functional magnetic resonance imaging data with corresponding DTI data may be needed to link the empirical data to the network models directly. Third, in the brain network models, it is difficult to generate ES network configurations that satisfy all ES conditions at the same time in the same levels observed in the empirical data. Therefore, in the model study, we focused on frequency difference and frequency assortativity, which were more robust ES conditions compared with the positive correlation between node degree and frequency. We implemented a V-shape relationship between frequency and node degree, which was suggested by Leyva *et al*.^19^ to produce similar levels of frequency difference and frequency assortativity with the results from the empirical data analysis. Fourth, the Kuramoto model is a simple coupled oscillatory model that was originally used for studying ES in model networks. For a more realistic simulation, a complex neural mass model that includes excitatory and inhibitory neuronal populations would improve upon the current brain network model of FM.

## Conclusion

The empirical data analysis demonstrated that pain in FM patients correlates with the strength of ES conditions in functional brain networks reconstructed from high-density EEG. Moreover, the human brain network model supported the hypothesis that network conditions for ES may give rise to hypersensitivity from a perturbation. The results suggest that ES can be a mechanism of a hypersensitive brain network in FM. Finally, we suggest that this could serve as a novel theoretical framework and quantitative approach to modulating chronic pain through the conversion of an ES brain network to a non-ES network using brain stimulation methods.

## Electronic supplementary material

Supplementary Information
