# Supplementary material for: Functional Brain Network Mechanism of Hypersensitivity in Chronic Pain
Source: Sci Rep. 2018 Jan 10;8:243. doi: 10.1038/s41598-017-18657-4 (PMC5762762; doi:10.1038/s41598-017-18657-4)
Supplement: Supplementary file 1 — Supplementary Information [file 41598_2017_18657_MOESM1_ESM.pdf]

Functional Brain Network Mechanism of Hypersensitivity in Chronic Pain

UnCheol Lee[1,2§], Minkyung Kim[1,5§], KyoungEun Lee[1§], Chelsea M. Kaplan[3], Daniel J. Clauw[1,4], Seunghwan Kim[5], George A. Mashour[1,2,3]*, Richard E. Harris[1,3,4]*

[1]Department of Anesthesiology, University of Michigan Medical School, Ann Arbor, MI 48109, USA

[2]Center for Consciousness Science, University of Michigan Medical School, Domino's Farms, P.O. Box 385, Ann Arbor, MI 48105, USA

[3] Neuroscience Graduate Program, University of Michigan, Ann Arbor, MI

[4] Chronic Pain and Fatigue Research Center, University of Michigan, Ann Arbor, MI 48105, USA

[5]Department of Physics, Pohang University of Science and Technology (POSTECH), Pohang, South Korea

* corresponding authors:
gmashour@med.umich.edu (G.A.M.), reharris@med.umich.edu (R.E.H.)

[§] these authors contributed equally to this work

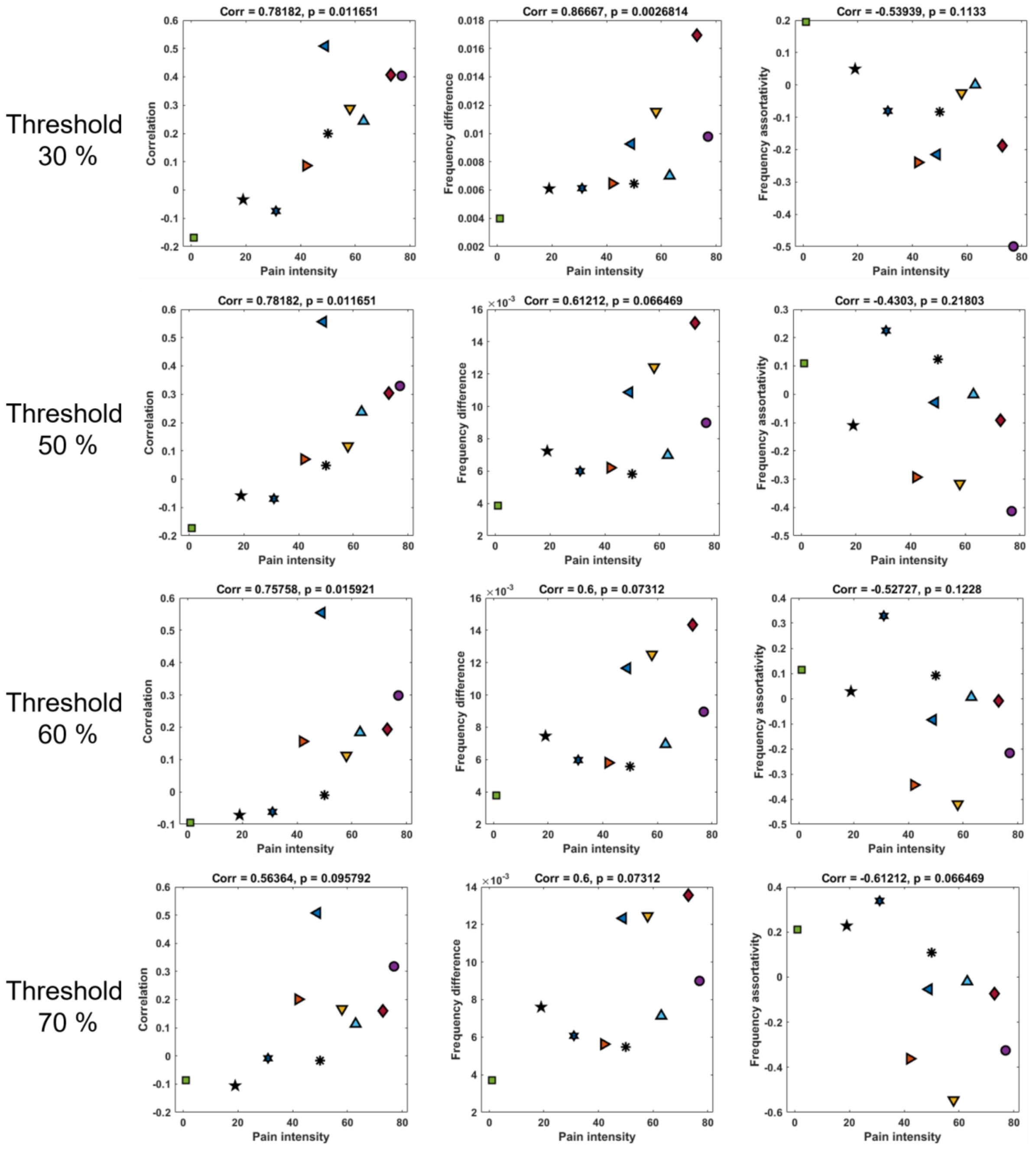

Figure S1. Three explosive synchronization conditions (Correlation between degree and frequency, frequency difference, frequency assortativity) for the threshold 30%, 50%, 60%, and 70%. A Spearman correlation and a p-value was calculated and written in the title of each figure. Below the 50% threshold our three ES conditions are consistent. We chose to report the 40% threshold for our analysis.

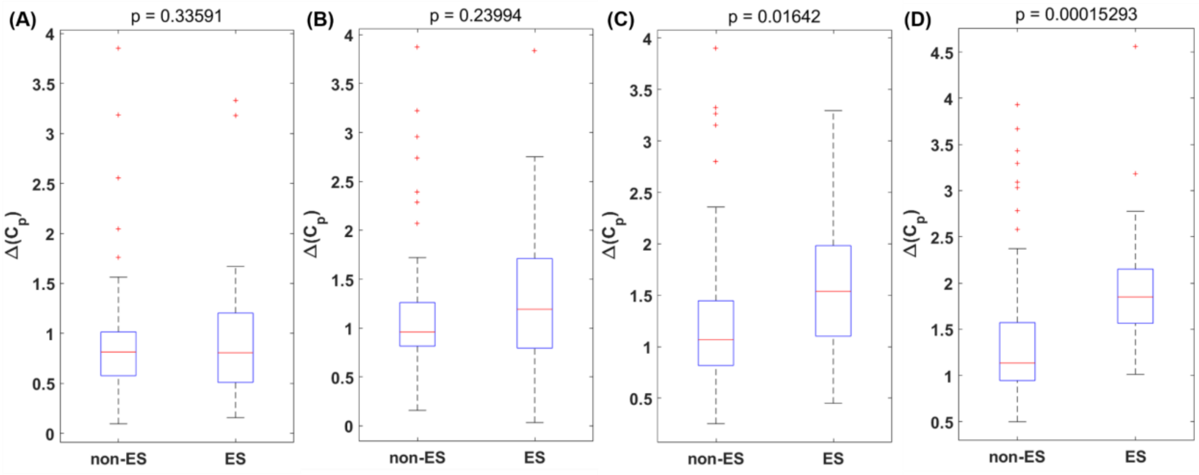

Figure S2. The network sensitivity of non-ES and ES networks with the various number of perturbed nodes. The largest degree nodes, (A) 1, (B) 5, (C) 10, and (D) 20 in the descending order are perturbed. In our simulation, the significant difference between non-ES and ES conditions is appeared from the perturbations to the top 10 degree nodes out of 82 nodes.

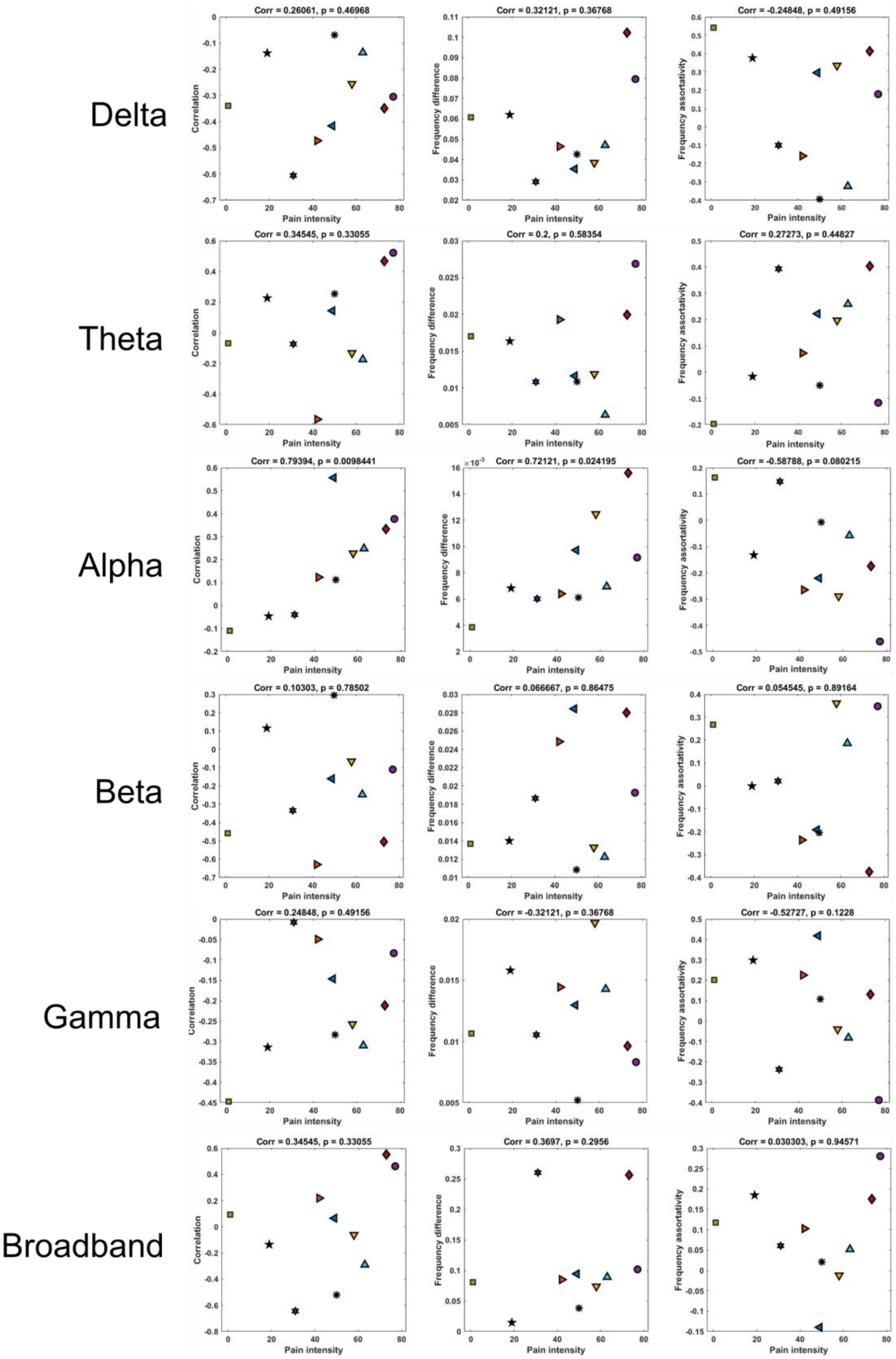

Figure S3. Three explosive synchronization conditions (Correlation between degree and frequency, frequency difference, frequency assortativity) for delta, theta, alpha, beta, gamma, and broadband (1-50Hz). A Spearman correlation and a p-value was calculated and written in the title of each figure. The results show only the alpha band has significant correlation coefficient for the pain intensity and the correlation between degree and frequency (0.79, $p<0.05$), and the correlation coefficient for the pain intensity and frequency difference (0.72, $p<0.05$). Otherwise, the others are not significant ($p>0.05$).

Supplementary Table S1. Names and abbreviations of brain regions.

	Region name
Subcortex	L. Thalamus
	R. Thalamus
	L. Caudate
	R. Caudate
	L. Putamen
	R. Putamen
	L. Pallidum
	R. Pallidum
	L. Hippocampus
	R. Hippocampus
	L. Amygdala
	R. Amygdala
	L. Accumbens
	R. Accumbens
Prefrontal	L. lateral orbitofrontal
	R. lateral orbitofrontal
	L. medial orbitofrontal
	R. medial orbitofrontal
	L. frontal pole
	R. frontal pole
Frontal	L. caudal middle frontal
	R. caudal middle frontal
	L. parsopercularis
	R. parsopercularis
	L. parsorbitalis
	R. parsorbitalis
	L. parstriangularis
	R. parstriangularis
	L. precentral
	R. precentral
	L. rostral middle frontal
	R. rostral middle frontal
	L. superior frontal
	R. superior frontal

**Central**	L. paracentral
	R. paracentral
	L. postcentral
	R. postcentral
**Temporal**	L. bankssts
	R. bankssts
	L. fusiform
	R. fusiform
	L. inferior temporal
	R. inferior temporal
	L. middle temporal
	R. middle temporal
	L. superior temporal
	R. superior temporal
	L. temporal pole
	R. temporal pole
	L. transverse temporal
	R. transverse temporal
**Parietal**	L. inferior parietal
	R. inferior parietal
	L. precuneus
	R. precuneus
	L. superior parietal
	R. superior parietal
	L. supramarginal
	R. supramarginal
**Occipital**	L. cuneus
	R. cuneus
	L. lateral occipital
	R. lateral occipital
	L. lingual
	R. lingual
	L. pericalcarine
	R. pericalcarine
	L. caudal anterior cingulate
	R. caudal anterior cingulate
	L. isthmus cingulate

**Cigulate cortex**	R. isthmus cingulate
	L. posterior cingulate
	R. posterior cingulate
	L. rostral anterior cingulate
	R. rostral anterior cingulate
**Parahippocampal cortex**	L. entorhinal
	R. entorhinal
	L. parahippocampal
	R. parahippocampal
**Insula**	L. insula
	R. insula
